# Enhancing Bioavailability of Nutraceutically Used Resveratrol and Other Stilbenoids

**DOI:** 10.3390/nu13093095

**Published:** 2021-09-02

**Authors:** Ondrej Vesely, Simona Baldovska, Adriana Kolesarova

**Affiliations:** 1 Department of Food Science, The Faculty of Agrobiology, Food and Natural Resources, The Czech University of Life Sciences Prague, 16500 Prague, Czech Republic; 2 AgroBioTech Research Centre, Slovak University of Agriculture in Nitra, 94976 Nitra, Slovakia; simona.baldovska@uniag.sk; 3 Department of Animal Physiology, Faculty of Biotechnology and Food Sciences, Slovak University of Agriculture in Nitra, 94976 Nitra, Slovakia

**Keywords:** resveratrol, bioenhancers, metabolism, bioavailability

## Abstract

Stilbenoids are interesting natural compounds with pleiotropic in vitro and in vivo activity. Their well-documented biological properties include anti-inflammatory effects, anticancer effects, effects on longevity, and many others. Therefore, they are nowadays commonly found in foods and dietary supplements, and used as a part of treatment strategy in various types of diseases. Bioactivity of stilbenoids strongly depends on different types of factors such as dosage, food composition, and synergistic effects with other plant secondary metabolites such as polyphenols or vitamins. In this review, we summarize the existing in vitro, in vivo, and clinical data from published studies addressing the optimization of bioavailability of stilbenoids. Stilbenoids face low bioavailability due to their chemical structure. This can be improved by the use of novel drug delivery systems or enhancers, which are discussed in this review. Current in vitro and in vivo evidence suggests that both approaches are very promising and increase the absorption of the original substance by several times. However, data from more clinical trials are required.

## 1. Introduction

Stilbenoids are part of a large group of phytochemicals known as polyphenols. Epidemiological studies have found an association between food consumption and health and longevity. Stilbenoids have become well known due to the compound resveratrol [1]. This molecule is well known for its association in explaining the lower mortality in France compared with the American population. This phytoalexin has helped to focus research on other similar compounds that are based on stilbene and slightly differ only in the numbers of hydroxyl groups, isomerization, glycosylation, styrene bond, methyl groups, prenyl groups, etc. The double bond is responsible for isometry, with the trans- form being more common from a steric point of view. Furthermore, isomerization has a strong influence on biological properties, and therefore it is important to use only the most potent variant in nutraceuticals [2,3,4,5]. 

Epidemiological studies have tried to find associations between food consumption and health effects in several ways [6]. There is a consensus that the intake of plant-based foods such as vegetables, fruits, nuts, grains, legumes, spices, roots, and leaves has an impact on health, especially in long-term and age-related diseases [7]. As the population ages, diseases such as cardiovascular disease, cancer, and type II and III diabetes become more common, especially in the countries that consume a Western diet [8,9]. The combination of aging and a significant shift in dietary habits from conventional foods to more convenient foods is probably behind the recommendations by a number of organizations such as the U.S. Department of Health and Human Services (DHHS) [10] and WHO/FAO, or country- and region-specific guidelines, namely, the Nordic Nutrition Recommendation, Pan American Health Organization (PAHO), and the Institute of Nutrition of Central America and Panama (INCAP) to incorporate more plants in the diet through the Food-Based Dietary Guidelines [11]. 

An alternative to plant-based diets are nutraceuticals that contain phytonutrients with biological properties, such as stilbenoids. These compounds are well studied for their anti-inflammatory properties [12], cardioprotective properties [13], and antidiabetic properties [14], all of which are related to age-related diseases. As in vivo experiments do not always correspond to in vitro experiments, more recent studies have focused on determining the effect of the low bioavailability of these compounds. 

The aim of this review is to summarize the pharmacokinetics of the best-known stilbenoid resveratrol and the approach to modifying its bioavailability.

## 2. Chemical Properties 

**Figure 1:**
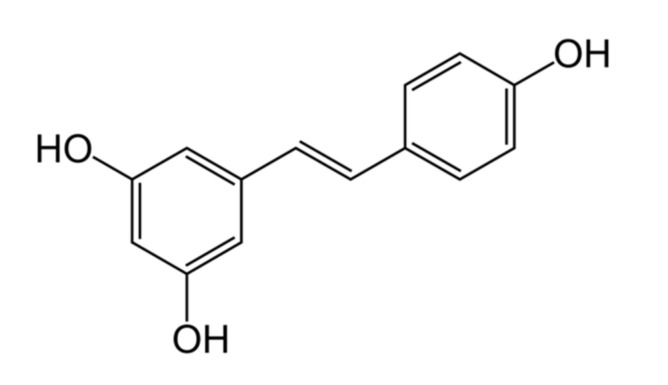
Chemical structure of *trans*-resveratrol.

Stilbenoids are naturally occurring phenolic substances, classified as phytoalexins, produced de novo in plants to protect against fungal infection and toxins and also to inhibit bacterial growth [15]. Resveratrol (3,5,4′-trihydroxy-trans-stilbene; RES, Figure 1), the most widely studied stilbenoid, is present in foods such as the skin of grapes, raspberries, blueberries, and peanuts [16]. RES has been known since its isolation from *Veratrum grandiflorum* by the Japanese scientist Michio Takaoka (of Imperial University of Hokkaido) in 1939 [17]. Its structure consists of two phenolic rings bonded together by a double styrene bond. This double bond is responsible for stereoisomerization, and thus two isomeric forms (cis- and trans-) are known, with the trans- form more common and more biologically active in comparison to cis- form [18]. On the other hand, another study showed the cis- form as a better inhibitor of tumor cells [19], so there is good evidence that isomerization of this molecule has an impact on the bioactivity.

The use of RES as a nutraceutical is limited due to its poor water solubility (<0.05 mg/mL), low oral bioavailability, and high chemical instability. In particular, the problem of low bioavailability of RES is crucial [20,21]. The absorption rate is approximately 75% (25 mg oral dose) [22,23], but it is questionable if this rate will be the same at higher doses. The absorption of RES by different types of cells was confirmed by several research groups [3,24,25,26]. Although the absorption is high, the bioavailability is poor [22]. RES is rapidly transformed by phase II metabolism. RES conjugates are rapidly absorbed through the gastrointestinal tract and are detectable in serum in 30–60 minutes after ingestion [22,23]. Moreover, there is evidence that the circadian rhythm can influence bioavailability [27] as well as the type of meal [28]. The time of ingestion of substances can strongly influence their bioavailability and rate of absorption. Questions regarding the ideal time of drug administration are addressed by chronopharmacokinetic studies [29]. To increase bioavailability, the best time to administer RES is in the morning [27]. The question of whether the intake of 400 mg of resveratrol can be influenced by a high-fat meal was investigated by Vaz-da-Silva et al. [30]. Their results showed that the rate of RES absorption was significantly delayed by the presence of food compared to fasting conditions. However, it did not have any significant effect on overall bioavailability. 

Important is the loading or dosage, as shown by another study [31]. RES concentrations vary significantly between studies, from nanomolar to millimolar, and dosage of more than 100 mg/day is considered important. Dose is a significant factor in the manifestation of the effect of RES, with smaller doses having a beneficial effect, while larger doses may be harmful [32]. Simply put, the tolerance to RES is cell-specific and varies depending on various organs; thus, it is difficult to define a safe dose limit. However, clinical studies suggest that relatively high doses of RES (2 g/day or more) begin to have non-life-threatening side effects such as diarrhea, nausea, and hypersensitivity [33], or frontal headache [27]. Finally, the functional and safe window of RES eventually lies somewhere between 100 and 1000 mg/day. 

## 3. Physiological and Therapeutic Effects

Stilbenoids possess numerous biological effects, including cardioprotective, neuroprotective, anti-inflammatory, and antidiabetic properties, and they can play a role in depigmentation, cancer prevention, and treatment [15]. RES exerts anticancer and antiproliferative, anti-aging, and antineoplastic activity in vitro and in vivo [34,35,36]. Antimicrobial activity has also been demonstrated against bacterial, viral, and fungal species, mainly in vitro [37]. Moreover, RES and other stilbenoids have well-studied anti-inflammatory properties in vitro and in vivo [12]. Inflammation is a process vital for survival and it is fundamentally a part of the defense against pathogens and serves as a response to tissue damage. It is normally recognized by immune cells (leukocytes, macrophages, mast cells, and neutrophils), and they release reparative agents at the site of inflammation, including cytokines, histamine, leukotrienes, nitric oxide, and prostaglandins. Three main pathways are known: NF-κB, MAPK, and JAK-STAT. As a result of inflammation, too many free oxygen radicals are produced. With prolonged inflammation, lipids, proteins, and nucleic acids can be damaged, which can lead to various types of diseases, such as atherosclerosis, cancer, inflammatory bowel disease, kidney diseases, lung diseases, liver diseases, and CNS diseases [38]. In chronic inflammation, RES is a promising regulator, protector, and therapeutic agent in the prevention of cardiovascular [39,40] and metabolic diseases, including obesity [40,41,42], as well as reproductive disorders [36,43] and reprotoxicity [44]. Furthermore, this bioactive molecule can extend the lifespan and ameliorate aging-related phenotypes, attenuate the negative effects of a high-calorie diet, mimicking the effects of calorie restriction, and improve cellular function and metabolic health in general [41]. Therefore, stilbenoids, such as RES, possess several physiological actions that can be useful for prevention, mitigation, and treatment of numerous diseases. Even small doses of RES can have an effect, as in the case of 10 mg RES, which significantly decreased insulin resistance via the Akt pathway, which led to improved insulin sensitivity in type II diabetes patients [45].

Although many studies describe the in vitro and in vivo modes of action of RES, some studies experimentally show the opposite action. For example, a study that tried to influence longevity in *Drosophila* failed [46]. In this case, the authors attributed the results to normal conditions. When Baur et al. attempted to prolong the life of rats, only those fed a high-fat diet had an effect [47]. These examples point to the influence of an individual’s health as a significant factor affecting the potency of RES. A meta-analysis of nine studies ( n = 208; RES dosage, 75–3000 mg/day; duration, minimum of 2 weeks) found no significant changes in body mass index or body weight [48]. As RES should activate adenosine monophosphate-activated protein kinase (AMPK) and thus induce a fasting state, weight loss would be expected. However, this has not been observed by all researchers. Different study results may be due to the differential period of dosing [49]. 

Thus, we can observe a significant influence of various factors such as age, health, duration and amount of the dose, and diet composition on the observed parameters. Since RES affects many cellular mechanisms, the methodology of the experiments needs to be adapted to the different mechanisms of action.

## 4. Mechanism of Actions 

RES can affect numerous cellular processes via multiple intracellular signaling pathways [50]. Mechanisms of actions include stress signaling pathway (MKP-1) and reduction of oxidative stress and inflammation (iNOS, COX-2 activity, and ROS generation), inflammation factors and malondialdehyde levels [51], as well as inhibition of prohypertrophic molecules, improvement of myocardial Ca^2+^ handling, and phosphorylation of prosurvival Akt-1 and GSK-3β [52]. Moreover, RES can prevent the expression of endothelial nitric oxide synthase and vascular endothelial growth factor, and suppress phosphorylation of p38 in rats with diabetes-related myocardial infarction. Additionally, the antioxidant activity of RES is associated with anti-aging effect and ability to affect apoptosis, growth, and carcinogenesis [36,43,53]. The binding of RES to various target molecules, which can be intracellular mediators of RES action, includes tubulin, protein kinase C alpha (PKCα), phosphodiesterase-4D [54], adenosine monophosphate kinase, nuclear factor-κB (NF-kB), inflammatory cytokines, antioxidant enzymes, and others [34]. Furthermore, RES can bind and affect DNA methyltransferases and proteins responsible for DNA methylation, which can be involved in epigenetic regulation of oxidative, metabolic, inflammatory, angiogenic, and tumorigenic processes [55].

RES is known as an activator of Sirtuin 1 (SIRT1), a NAD^+^-dependent histone deacetylase contributing to oxidative stress, aging, metabolism, obesity, and tumors, as well as to DNA methylation [50]. A meta-analysis of 11 randomized controlled trials found a significant influence of RES as a treatment for diabetic parameters such as fasting glucose, insulin, glycated hemoglobin, and insulin resistance in patients with diabetes mellitus (DM) type II [56]. Interestingly, these results are not seen in patients without comorbidities. Researchers have discussed mainly the following mechanisms. First, RES is known as an activator of SIRT 1, thus mimicking the caloric restriction and providing a beneficial effect on glucose control [57,58]. Secondly, RES can increase the expression of the insulin-regulated glucose transporter protein (GLUT4), which is an important factor for modulating insulin resistance [59,60] and improving glucose uptake in the absence of insulin [61]. Because RES can directly alter the secretion of insulin by the pancreas, it may explain the large effect on DM II [62]. All these mechanisms of action can influence a wide array of cellular processes via multiple intracellular signaling pathways.

## 5. Pharmacokinetic Characteristics of Stilbenoids

Because the efficacy of orally administrated stilbenoids depends on their absorption, metabolism, and tissue distribution, it is important to understand their overall pathway. 

### 5.1. Pharmacokinetics in Animals

To our knowledge, the first study in rats demonstrated absorption of RES from the gastrointestinal tract way back in 1996 [63]. In their experiment, Bertelli et al. added known RES content (6.5 mg/L) in the form of red wine. The results of the study showed that the RES contained in the wine was rapidly absorbed and was detectable within 30 min, peaking at approximately 60 min after ingestion of the wine [64]. 

Another study in rats [65] described the rapid absorption of RES in blood and serum with a calculated absorption at 50–75%. Moreover, the type of matrix (ethanol, V-8 vegetable cocktail, and white grape juice) did not increase absorption. In addition, trace amounts in the liver, kidney, heart, and spleen were found. Another study confirmed these results in mice but found RES at high concentration in urine and bile [66]. Three hours after administration, RES was detectable in various organs, with the highest concentration in the duodenum, lower in the kidneys, followed by the lungs and liver. In rats, RES is metabolized in the liver by cytochrome P450. Common metabolites are *trans*-resveratrol-3-O-glucuronide and *trans*-resveratrol-3-O-sulphate [67]. In another study, differences between dosage and metabolites were found, and higher dosage (300 mg/kg body weight) led to further of *trans*-resveratrol-3,4’,5-trisulphate [68]. These were detectable in plasma, liver, and kidney samples.

The animal model close to human is pig [69]. In this study, twelve resveratrol and seven dihydroresveratrol metabolites, all of them close to those of previous studies (*trans*- and *cis*-resveratrol, nine resveratrol conjugates such as glucuronide, sulphate, and sulphoglucuronide derivatives), the microbiota-derived metabolite dihydroresveratrol, and six conjugates (also glucuronide, sulphate, and sulphoglucuronide derivatives) were detected. Approximately 74.5% (351.6 mg) of the total RES (472 mg) administered was recovered in the form of RES, dihydroresveratrol, and derived metabolites (65.1% along the gastrointestinal tract, 7.7% in urine, 1.2% in bile, and 0.5% in organs). The authors considered that the results obtained may vary depending on the time of sampling. In their study, all the data were obtained six hours after administration of RES.

In summary, RES in animals is absorbed in the intestines, then converted to several metabolites, mainly glucuronide and sulphate, and finally excreted, mainly in the urine and stool. For a more precise approach, it is important to move from animal to clinical evidence. 

### 5.2. Pharmacokinetics in Humans

There are studies on the pharmacokinetics of RES in humans. Goldberg et al. [23] followed up on a previous study [65]. *Trans*-resveratrol (25 mg/70 kg) was administered in men at four-week intervals in three different matrices: white wine (11.5% ethanol), grape juice, and vegetable juice. RES was present in serum and urine predominantly as glucuronide and sulphate conjugates, reaching peak concentrations approximately 30 min after consumption. Free RES accounted for 1.7% to 1.9%. The study demonstrated human ability to metabolize RES that was not affected by any type of matrix used [23]. A more accurate study used radioactive RES (^14^C RES) both orally (25 mg (110 µM)) and intravesically (0.2 mg) [22]. Most of the radioactivity after the oral dose was recovered in the urine (53–85%). The recovery in stool was highly variable (0.3–38%). The research group also compared oral dose with the i.v. dose of 0.2 mg; the recovery in urine was 42–83% and in stool 0.6–23%. One volunteer was exposed orally to 100 mg of an unlabeled dose of RES to determine the structure of RES metabolites. Five major metabolites, two monoglucuronides, dihydroresveratrol monoglucuronide, resveratrol monosulphate, and dihydroresveratrol sulphate, were detected in urine. After oral administration, 24% ± 3% of the sulphate conjugates and 13% ± 1% of the glucuronic acid conjugated were excreted in the urine. This study showed that RES is rapidly metabolized and absorbed in humans after consumption. Only 2% free RES can be detected in plasma and serum [22]. 

The two main reactions for the formations of metabolites in humans are glucuronidation and sulphation. The UDP-glucuronosyltransferase (UGT) family catalyzes the conjugation of RES to glucuronic acid at the 3’ or 4’ hydroxyl group. These enzymes are abundant in intestinal microsomes and in the liver. UGT1A1 and UGT1A9 were found to be predominantly responsible for the formation of 3-O-glucuronide (K_m_ = 149 µM) and 4’-O-glucuronide (K_m_ = 365 µM), respectively [70]. Remarkably, intestinal glucuronide conjugates formed up to 10-fold more compared to the hepatic microsomes. The second pathway commonly used by the body to eliminate polyphenols is sulphation. In the liver, RES is metabolized by sulphotransferase (SULT), where resveratrol-4’-O-sulphate is formed by SULT1E1, and resveratrol-3-O-sulphate is formed by either SULT1A2 or SULT1A3 [71]. In summary, 75% of RES passes through the apical and basolateral membranes by simple diffusion. In cells, RES undergoes rapid and extensive modification by the enzymes UGT and SULT, resulting in glucuronide and sulphate conjugates. The metabolites leave the cell via multidrug resistance protein (MRP 3—basolateral side) or breast cancer resistance protein (BRCP) and MRP2 (apical side) [72,73].

The microbiota plays an important role. The most common metabolites of the human gut microbiota are dihydroresveratrol, 3,4’- dihydroxy-*trans*-stilbene and lunularin [74]. In the same study, Bode et al. identified two bacterial strains, *Slackia equolifaciens* and *Adlercreutzia equolifaciens*, as dihydroresveratrol producers, which means that the composition of microbiota influences the resulting metabolites. When Jarosova et al. attempted to investigate multiple stilbenoids in a similar manner to that of Bode et al., their findings showed dihydroresveratrol as the only one bacterial metabolite [75]. Jose et al. discovered an effect of RES and other stilbenoids on an abundance of specific taxon, *Faecalibacterium prausnitzii*, which they consider beneficial [76]. This evidence points to the possibility of a prophylactic effect of these substances and the subsequent formation of other metabolites and influence on pharmacokinetics.

**Figure 2:**
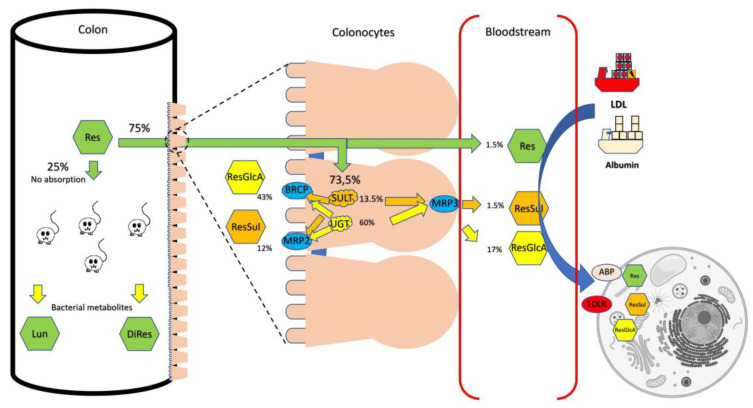
When resveratrol (RES) reaches the colon, approximately 75% can enter the colonocytes and the remainder can be metabolized by the microbiota into metabolites such as dihydroresveratrol (DiRes) or lunularin (Lun). These may enter the cells or be excreted with the remaining resveratrol in the feces. Colonocytes can convert RES into metabolites via sulphotransferase (SULT) or UDP-glucuronosyltransferase (UGT). These are pumped mainly apically to the lumen via BRCP (breast cancer resistance protein) and MRP2 (multidrug resistance protein) and partly into the circulation via MRP3. When RES or metabolites pass through the cells, they are absorbed by low-density lipoprotein (LDL) or albumin and distributed throughout the body. When they reach the cells, they can be released via LDL-receptor (LDLR) and albumin binding domain (ABP) and subsequently absorbed by the cells. Finally, the cells can deconjugate them.

Once stilbenoids enter the circulation, they must be transported. In the bloodstream, RES and its metabolites can bind to plasma lipoprotein (LDL) and albumin and thus be transported between tissues. These complexes can be dissociated on cell membranes, leaving RES free to enter cells [77,78]. Most importantly, there is evidence that metabolites of RES can be deconjugated back to RES [79]. In this study, a human aortic endothelial cell line (HAEC) and 0.5–50 µM RES were used. This dose is similar to those obtained after pharmacological intake. However, there is still not enough information about stilbenoids and different cell types, but, as a similar pattern can be observed for other polyphenols, it is likely that this also applies to stilbenoids [80]. The fate of orally ingested RES is shown in Figure 2.

In the end, the largest amount of RES, exceeding the amount required for in vitro activity, can be detected in colon tissues, so the colon can be considered the main target organ. The efficacy in other tissues may depend on its metabolites and the ability to regenerate RES locally or systemically [81].

## 6. Enhancing the Bioavailability of Stilbenoids

The bioavailability of drugs can be influenced by many factors, namely, effect of pH, acid secretion, delayed gastric emptying, and enzyme activity by the stomach, the release of bile salts by gall bladder, secretion of enzymes into the small intestine and production and secretion of insulin by pancreas, bile salt concentrations, enzyme activity, viscosity and osmolality, potential inhibition of transporters and drug-metabolizing enzymes, amd potential changes in lymphatic uptake by small intestine and colon [82]. The important factor is the food matrix [83]. Proteins, dietary fiber, and minerals can negatively alter the bioavailability of flavonoids, and on the other side, the lipids, digestible carbohydrates, vitamins, alkaloids, carotenoids, etc. are likely to improve flavonoid bioavailability [83]. As flavonoids have a similar molecular structure to that of stilbenoids, we can assume similarities. Moreover, there are ways to increase the bioavailability of stilbenoids, namely, using bioenhancers and novel methods.

### 6.1. Bioenhancers

**Figure 3:**
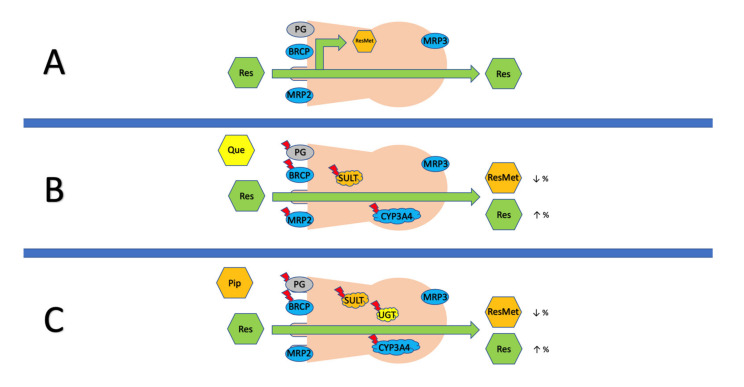
Increasing intestinal permeability with (**A**) RES alone, (**B**) quercetin, and (**C**) piperine. As shown in row, RES (**A**) diffuses into cells without active assistance, is metabolized by SULT and UGT enzymes to metabolites (ResMet), and only a small volume reaches the basolateral side. Quercetin (**B**), when added to RES, inhibits ABC transporters (specifically PG, BRCP, and MRP2) and SULT. This combination leads to a decrease in sulphate metabolites and an increase in RES on the basolateral side. If piperine (**C**) is added, there are not enough studies against MRP, but like quercetin, it is able to inhibit ABC transporters, SULT, and even UGT. This leads to a decrease in sulphate and glucuronide metabolites and an increase in RES.

Bioenhancers promote and augment the bioavailability of compounds without having any activity with the drug of their own at the dose used. For example, alkaloid piperine has a molecular structure suitable for enzyme inhibition. Piperine, which is a potent inhibitor of drug metabolism, is therefore a strong enhancer of bioavailability. Piperine works through increased gastrointestinal absorption (enhancing solubility, increased blood supply, increased permeability due to epithelial cell modification, through increased microvilli length), reduction of efflux of drugs from the site of action, inhibition of solubilizer attachment, and metabolism reduction [84]. The in vitro, animal, and interventional studies involving stilbenoids and bioenhancers are summarized in Table 1. In vitro experiments using a permeability model show a significant effect of enhancers such as quercetin or piperine. These results are consistent with experiments in animal models. In contrast, they are difficult to apply to results from clinical studies. However, there is still not enough evidence of whether increased bioavailability leads to increased bioefficacy of the target compounds. Wightman et al. used a combination of 250 mg of RES and 20 mg of piperine as bioenhancer. Contrary to in vitro and animal studies, the Cmax concentration of RES alone was 9.98 µM, while with piperine it was only 4.82 µM. Based on results, researchers concluded that co-supplementation of piperine with RES can enhance the bioefficacy of RES with regard to effects on cerebral blood flow in healthy human subjects, and does this without altering the bioavailability of RES, since the plasma concentrations of RES and its metabolites were not different between the treatments [85]. In another study, the combination of 2 g RES and 500 mg quercetin two times a day was used [28]. The combination did not affect the pharmacokinetics of RES. With the current number of studies and the limited number of substances tested, it is not clearly understood as to why the results vary so much at different levels of research. For example, it is possible that although substances inhibit a particular metabolic pathway, probably another persists. Another possibility could be that, due to the complexity of the organism, the enhancer is added in insufficient amounts. More evidence is needed. A simplified operation of the best-known bioenhancers can be seen in Figure 3.

Interestingly, in mice, RES can enhance other biologically active compounds such as apigenin [91]. As resveratrol–apigenin shows, stilbenoids can alter the bioavailability of different compounds. For example, RES, which is generally accepted to be a moderate-to-weak inhibitor of cytochrome P450 (CYPs), is known as an inhibitor of CYP3A4, but inhibition of these enzymes by RES aglycons is not known, although they are more abundant in the body than RES themselves due to metabolism [92]. Because CYP3A is common in small intestine, stilbenoids can alter the first pass metabolism [93]. They also can affect the efflux transporter p-glycoprotein. This ATP-binding cassette transporter (ABC transporter) plays an important role in drug transport in many organs. For example, in the intestine, p-glycoprotein pumps drugs back into the lumen, leading to reduced absorption. P-glycoprotein transporters are more abundant in cancer cells, where they influence survival against anticancer treatment [94]. This transporter is also common in small intestine, liver, and kidney as well as part of the blood brain barrier or placenta [95]. An interesting study using C57BL/6J mice as an animal model combined RES and an alkaloid berberine, which is known for its antidiabetes properties [95]. In this case, the combination of the two agents had a significantly greater effect on cholesterol and low-density lipoprotein cholesterol (27% and 31%, respectively) compared with monotherapy with RES alone (8% and 6%, respectively) and berberine (10% and 10%, respectively). Moreover, RES was able to increase the amount of berberine intracellularly in L02 liver cells. Several studies show that RES can break multidrug resistance by altering p-glycoprotein, and therefore it is better to look at stilbenoids not only as main drugs but also as supportive drugs [96,97,98,99].

### 6.2. Novel Methods

Novel methods or novel drug delivery systems (NDDSs) are a way to deliver the maximum amount of drug to the target site. This can alter the duration of therapeutic activity and the bioavailability [100]. Stilbenoids can be delivered by different types of systems [101], namely, microspheres [102], microcapsules [103], self-emulsifying drug delivery system (SEDDS) [101,104], self-microemulsifying drug delivery system (SMEDDS) [105,106,107], nanosponges [108], nanoemulsions [109,110], self-nanoemulsifying drug delivery system (SNEDDS) [111,112,113,114], self-double-emulsifying drug delivery system (SDEDDS) [115], liposomes [116,117], nanoparticles [118,119,120,121,122,123,124,125,126], nanostructured lipid carriers [127], nanosuspensions [128], chitosan–gellan nanofiber [129], solidified phospholipid complex [130], spiral dextrin [131], and buccal cyclodextrin delivery system [132]. All delivery systems are extensively studied, but there is little clinical data. Novel methods are more closely discussed in a recent review by Chen et al. [133]. However, more clinical data are needed.

Few clinical studies are known to examine novel methods on humans. In the study by Calvo-Castro et al., a group of 12 subjects were given a single dose of vineatrol (30 mg RES and 75 mg viniferin) in powder or liquid micellar formulation. The maximum total RES concentrations in plasma were 10.6-fold higher when ingesting micellar compared with native vineatrol [134]. Briskey and Rao randomized 39 subjects into four groups and used a novel dispersion agent for screening to enhance RES absorption. The data showed a 2-fold increase in plasma concentration over 24 h and a 3-fold increase in Cmax of both the sulphate and glucuronide conjugates [135]. 

In vitro, animal, and clinical studies using novel methods to improve stilbenoids uptake promise a significant advance in bioavailability. However, there are currently insufficient studies to confirm and compare the clinical efficacy of different approaches.

## 7. Conclusions

In summary, stilbenoids exhibit many biological properties both in vitro and in vivo. However, due to their low solubility and bioavailability, clinical studies often do not correspond to in vitro studies. We have shown that stilbenoids are mostly metabolized in the human body to glucuronide and sulphate conjugates in the intestine and liver. These metabolites do not have similar biological properties and therefore, new delivery systems have been developed to improve bioavailability and new research has been conducted on enhancers. Only a few enhancers are known, mainly piperine, but it has not been successful in clinical trials to date. The influence of the food matrix is important for polyphenols, but data are lacking in the stilbenoids. 

Finally, we can conclude that there are several promising approaches to increase the bioavailability of stilbenoids, but to date, there is insufficient evidence from human studies, and further research is required. However, recent data suggest that a combination of enhancers and novel drug delivery systems can be very effective.

## Figures and Tables

**Table 1:** Summary of the in vitro, animal, and interventional studies involving stilbenoids and bioenhancers.

Compound	Dosage	Effect	Type	Animal/Cell	Results	Reference
Resveratrol	5 g per person	Peak serum concentration 539 ng/mL RES Metabolites 3-8× higher compared to RES	Clinical studies		Low bioavailability	[20]
Resveratrol + piperine	250 mg RES vs. 250 mg RES + 20 mg PIP	No vs. Increase cerebral blood flow			No altering bioavailability	[85]
Resveratrol + piperine	2.5 g RES + 0; 5 or 25 mg PIP	No relationship between dose and pharmacokinetic values			Dosage does not alter bioavailability	[86]
Resveratrol + quercetin + alcohol	2 g 2×/person + 500 mg Q + 5 mL alcohol	Neither quercetin nor alcohol alter RES. Fat meal alter RES concentration			Fat meal decreases bioavailability	[28]
Resveratrol + piperine	100 mg/kg RES vs. 100 mg/kg RES + 10 mg/kg PIP (oral)	After comparison maximum serum concentration increased to 1544%	Animal studies	C57BI/6 mice	Beneficial	[87]
Resveratrol + piperine	30 mg/kg	RES with piperine loaded mixed micelles demonstrated 5.7-fold compared to RES		Wistar albino	Beneficial	[88]
Oxyresveratrol + piperine	100 mg/kg OXY vs. 100 mg/kg OXY + 10 mg/kg PIP (oral)	Piperine mix increased bioavailability of oxyresveratrol 2-fold		Wistar rat	Beneficial	[89]
Resveratrol + curcumin + quercetin + piperine	50 µM (RES, C, Q)200 nM (PIP)	RES received the greatest enhancement in permeability when combined with other agents: quercetin (310%), curcumin (300%), quercetin and curcumin (323%, 350% with piperine). Curcumin also demonstrated increased permeability when combined with quercetin alone (147%) and both quercetin and resveratrol (188%), addition of piperine resulted in a 229% increase in permeability.	In vitro/ex vivo studies	Caco-2	Beneficial	[90]

RES: resveratrol, PIP: piperine, OXY: oxyresveratrol, Q: quercetin, C: curcumin.

## Data Availability

No new data were created or analyzed in this study. Data sharing is not applicable to this article.
